# Collaborative Preclinical/Clinical Efforts Aimed at Strengthening a Neurology Residency Program: Challenges, Activities and Outcomes

**DOI:** 10.1007/s40670-024-02192-9

**Published:** 2024-10-15

**Authors:** Michael F. Nolan, John P. McNamara

**Affiliations:** Virginia Tech Carilion School of Medicine, 1 Riverside Circle, Suite 202, Roanoke, VA 24016 USA

**Keywords:** Content integration, Biomedical sciences, Residency education, Faculty benefits

## Abstract

Recent trends in medical education have included efforts to better integrate traditionally preclinical content with subject matter included in the clinical years of the curriculum. The participation of clinical faculty in preclinical courses is well established; however, involvement of biomedical science faculty in resident education is less common. We describe here a project in which a basic science faculty member participated in a neurology residency program to address specific basic science knowledge weaknesses identified by the neurology department chair. We address issues and challenges associated with planning and implementation. Benefits to both the residents and the biomedical scientist are described.

## Introduction

The education and training of physicians require a faculty with wide-ranging experience and expertise in both the basic and clinical sciences. These requirements apply to both undergraduate medical education (UGME) and graduate medical education (GME) programs. The increasingly rapid advances in the biomedical sciences have prompted wide-ranging changes in medical curricula in recent years. Curricular revisions have involved deletions, additions, and shifts in emphasis as well as changes in teaching and assessment approaches.

Among the challenges faced by administrators and curriculum developers is how to best incorporate these new content areas and teaching methods in settings marked by desires to reduce scheduled curricular time and constraints related to fiscal and other resource availability. The progressive increase in the volume and complexity of information now being included in contemporary curricula highlights the need for appropriately trained faculty to develop approaches in ways that are efficient and effective.

Approaches in the UGME environment include efforts that intentionally integrate the traditional preclinical and clinical components of the curriculum [1–4]. Most commonly, these efforts involve incorporating clinically important content into preclinical courses [5, 6]. Others have described efforts in which basic science content is integrated into clerkship experiences and elective courses [7–10]. Clay et al. [11] described an effort in which basic science educators attended selected patient care activities and hospital rounds as observers to gain insights into clinical education practices and to contribute basic science correlations as appropriate. These authors reported a number of benefits including the development of a better understanding of the student experience in the clinical years and suggested that these types of interactions might also foster new collaborative efforts in areas of interest other than education [11].

The integration of basic science content into GME programs is another area of interest and activity. Lectures on specific basic science topics are not uncommonly included in the didactic components of a residency program. In some surgical specialties, residents return to the dissecting laboratory or surgical skill laboratory to review the anatomy of particular regions or to practice various surgical procedures on appropriately prepared cadaver material.

We describe here an effort involving a clinical department (neurology) and a basic science department (anatomy) to address a specific concern identified by the neurology department chair, specifically resident difficulties in describing and explaining the neuroanatomical bases of various tests and maneuvers used in the neurological examination. We describe the efforts undertaken in the planning and development of focused learning activities designed to remedy these deficiencies. We outline the instructional components of the program developed for this purpose and summarize benefits accruing to the residents, the residency program and the participating faculty, both basic and clinical. We include data documenting improvement in resident neuroanatomical understanding over time and provide subjective, narrative comments from residents and faculty regarding their perceptions of the program. Lastly, we call attention to factors to consider when developing such programs to ensure success.

### Program Planning and Development

#### Problem Identification

As part of an overall neurology departmental self-study and review, concerns were raised regarding residents’ ability to describe neuroanatomical structures, pathways, and functions that were being evaluated when performing the neurologic examination. Residents had difficulty identifying pertinent neuroanatomical structures and pathways, explaining neurophysiological mechanisms associated with particular tests and maneuvers, and explaining the mechanism of certain abnormal examination findings such as the crossed adductor reflex and ocular deviation observed in patients with brainstem or unilateral supratentorial lesions. These deficiencies contributed to difficulty in being able to precisely localize certain lesions and arrive at a complete and reliable differential diagnosis. Difficulties with application of previously learned basic science information are not unique to neurology. Similar difficulties in the ability to recall and apply previously learned information during general internal medicine clerkship rotations have been reported [12, 13].

#### Initial Planning and Developmental Activities

In an effort to address these concerns, the Chair of the Department of Neurology contacted the Chair of the Anatomy Department to discuss possible ways to solve these problems. A meeting was scheduled involving the two chairs, the neurology department program director and an individual from the anatomy department who had interest and experience in teaching neuroanatomy.

The planning and development process was begun approximately 9 months before the beginning of the resident academic year: the second week in July. The planning meetings involved the neurology program director and the identified anatomy department faculty member. The meetings addressed specific needs and goals and an outline for a formal series of learning activities was developed. The plan developed included session content and materials to be developed, methods of delivery, placement of the learning activities in the residency program, and a method to evaluate the success of the learning activities. Other matters including time commitment of participating faculty and possible costs were addressed. Approval of the overall goals and approaches was obtained from the department chairs and work was begun with the goal of implementation with the incoming residency class.

In an effort to become better acquainted with the neurological examination, the anatomy faculty member, a Ph.D. neuroanatomist and Physical Therapist, attended weekly neurology grand rounds, ward rounds on a regular basis, and various specialty clinics to gain a better understanding of routine resident responsibilities and activities. Attention was focused on identifying basic neuroscience content that was important, if not essential, in performing and interpretating neurologic examination findings. Meetings involving the neuroanatomy instructor and the neurology program director were held to define the scope and depth of included content and topics and to create a workable class schedule. The learning activities were to include didactic lectures, applied neuroanatomy exercises, and formal written assessments. The series of sessions was limited to topics identified as important in terms of the specific needs as described by the Department of Neurology Chair and Program Director (Table 1).

**Table 1:** Lecture and applied neuroanatomy session outline

Mental Status (MMSE)
Level of consciousness (Glasgow coma scale/lethargy/obtundation/stupor/coma) Attention (spell WORLD backward/recite months of year backward) Orientation (description of person, place, time, situation) Language (comprehension/fluency/repetition/reading/writing/naming-word finding/prosody) Cortical and Cognitive (fund of knowledge/planning/executive function/apraxia/calculation/proverb interpretation) Memory (immediate recall/short term/long term/anterograde/retrograde) Thought Content (mood/affect/delusions/hallucinations/illusions/phobias/paranoia/coherency)
Sensory Systems
Anterolateral system (pain/thermal testing) Lemniscal system (light touch/vibration/position sense) Cortical sensory functions (agnosia/traced figure ID/object ID/extinction)
Motor Systems
Lower motor neurons (strength testing/paresis and paralysis/atrophy) Upper motor neurons (weakness/spasticity/laterality) Basal nuclei (akinesia/rigidity/rest tremor/postural stability/chorea/athetosis ballismus/tics/hypomimia) Cerebellum (dysmetria/dysdiadochokinesia/intention tremor)
Reflexes
Muscle tone (hyper and hypotonia/dystonia/clonus) Muscle stretch (threshold/latency/amplitude/spread/duration) Cutaneous (plantar/withdrawal/abdominal/anal) Developmental (Moro/Landau/ASTNR/TNR)
Cranial Nerves
Olfactory (anosmia/hallucinations) Optic (fields/acuity/optic disc and macula/ophthalmoscopy — papilledema/atrophy/scotomas/cataracts) Oculomotor nerves (diplopia/tropia/phoria/anisocoria/ptosis/cover testing Adie’s pupil/Marcus-Gunn pupil/red glass and Maddox rod tests) Trigeminal (sensory/motor/trigeminal reflexes/dysarthria) Facial (facial expression/taste/lacrimation/facial reflexes/dysarthria) Vestibular (nystagmus/vertigo/syncope/unsteadiness/Epley/Dix-Hallpike) Cochlear (screening maneuvers/hypacusis/tinnitus/Rinne/Weber) Glossopharyngeal and Vagus (gag reflex) Spinal Accessory (strength testing/torticollis) Hypoglossal (deviation/atrophy/dysarthria)

### Program Description

The didactic component would include a review of basic neuroanatomy and neurophysiology with a focus on structures, principles, relationships, and mechanisms that were needed to correctly perform and reliably interpret findings elicited on the neurological examination. The sessions would include formal lectures and application exercises in which residents would have the opportunity to demonstrate, that is, apply their knowledge and understanding of neuroanatomical principles in the performance of specific neurological examination tests using their peers as subjects.

The applied neuroanatomy exercises were designed as interactive sessions overseen by the faculty in which residents, using each other as subjects, performed maneuvers and techniques associated with the neurologic examination. During these sessions, residents were asked to describe the neural structures, pathways, and physiological mechanisms being demonstrated. Faculty assistance was provided when deficiencies in understanding or application were noted. Diagnostic inferences and pathological mechanisms associated with possible abnormal findings were also discussed during these sessions.

The applied neuroanatomy activities occurred on the same day a topic was considered in the lecture form. Since many of the activities involved particular examination techniques and diagnostic tools, advantage was taken to ensure that the maneuvers were being performed properly and the diagnostic tools were being used correctly. For most sessions, the neurology program director or other members of the neurology department faculty participated to ensure the proper performance of these clinical exam-like activities.

A syllabus was created which included session outlines with objectives, useful images, and recommended readings. An applied neuroanatomy guide was developed that described the applied anatomy exercises and included practice questions related to neuroanatomical and neurophysiological principles associated with the neurological examination. The sessions were considered mandatory and a schedule was developed that allowed for all residents to attend without affecting their patient care responsibilities.

Class sessions were 90 min in length and scheduled weekly beginning in July, shortly after the arrival and in-processing of new residents. Sessions were held in a room in the university clinic used for educational purposes. Each of the 28 sessions was sharply focused on neuroanatomical and neurophysiological principles needed to perform, interpret, and understand particular clinical tests and their expected results. Residents in all 3 years of training were required to attend the sessions.

A written examination of 50 multiple-choice questions dealing with the topics considered was developed and administered to all residents during the first session in July of each of academic year and repeated during the first session of the following year. Test results were reviewed by the residency program director, the department chair, and the individual resident. Progress was noted and areas in need of strengthening were identified. The examinations were identified as a formal component of the resident training program and results were included in documentation used for program re-accreditation purposes.

Formal feedback from the residents was obtained each year following administration of the annual written examination. Residents were encouraged to indicate whether the sessions provided new information or contributed to a better understanding of their existing neuroanatomical knowledge and if and to what extent the information in the sessions helped them in any way in performing or interpreting the neurologic examination. Feedback was also solicited from the faculty twice, once at the end of the academic year and again at the midway point. Faculty were asked to comment on any changes they might have observed in resident ability to perform or interpret neurologic examination findings or in drawing correct conclusions from the examination data.

### Outcomes and Benefits

#### Written Examinations

Written examinations’ scores showed progressive improvement over time as residents progressed through the program. Mean scores of all residents taking the examination at the beginning of their PGY 1, 2, and 3 years were 63%, 74%, and 87%, respectively. A mean score of 89% was recorded for all residents at the end of their residency training. An expected upward trend in examination scores was observed for all residents participating in learning activities. However, we interpret these data with caution, recognizing that a number of factors ranging from experience gained through clinical practice to individual study and review of basic science information undoubtedly contributed to this finding. Nonetheless, performance on the annual written examinations provided useful feedback to the individual resident, identifying weaknesses and in guiding independent study.

Data from the written examination and feedback during the applied neuroanatomy sessions aided the neurology program director in both recognizing excellence in performance and in being able to design and initiate timely and appropriate remediation strategies when necessary. In several cases, specifically for residents in the early years of residency training, difficulties in interpreting specific findings elicited on clinical examination could be correlated with weaknesses in neuroanatomical knowledge. Recognition of these focal knowledge gaps allowed for focused efforts aimed at improving resident performance over time.

#### Department of Neurology and Residents

For the neurology department, a description of the didactic content and the objective data regarding resident performance was included in materials assembled for purposes of program re-accreditation. These data addressed the issue of a sufficiency of didactic content within the residency program and provided supplementary evidence regarding resident achievement and performance over time. A description of this program is included during elective rotations for third- and fourth-year medical students and during rotations by students from other schools in an effort to recruit resident applicants who might be considering applying to our school.

#### Anatomy Department and Basic Science Faculty Participant

The opportunity throughout the project for the basic science faculty member to attend rounds and clinics resulted in a greater understanding of clinical neurology and appreciation of how basic science material is used in clinical settings. Insights were gained regarding specific content areas that warranted particular attention in the preclinical neuroscience course which he directed. This experience provided important guidance in modifying preclinical instruction, adding or deleting topics, and adjusting emphasis to enhance the value of the preclinical course.

Insights gained also contributed to the development of new teaching materials to supplement or replace previous materials. Specifically, the applied neuroanatomy activities and the lecture notes were modified for use in a preclinical basic science course. Several of these work products were submitted for peer review and have been published [14–16].

The efforts described here have led to other collaborative efforts between faculty of the two departments. These included contributions as co-author on papers and presentations at national meetings and as co-investigators on selected research projects.

#### Themes Emerging from Residents’ Comments

Narrative comments were solicited from the residents at the end of the final class session of the year immediately following administration of the written examination. All residents submitted narrative comments. Four themes emerged from the received comments:

1. The sessions clarified concepts, most of which they were familiar with from their UGME experience, but about which they were uncertain.
2. Residents appreciated the hands-on “application activities.” These allowed for supervised practice of common neurologic examination tests with feedback that improved performance and understanding. Also appreciated was the opportunity to learn about and practice with less commonly used diagnostic tools including (e.g., Maddox rod).
3. Session content was thought to be transferable to the clinical setting and contributed to confidence in interpreting examination findings.
4. Sessions were viewed as an overall beneficial utilization of curricular time.

#### Themes Emerging from Faculty Comments

Narrative comments were solicited from neurology faculty twice, once at the midway point of the academic year (December) and again at the end of the academic year (June). Faculty were asked to comment on changes observed in individual residents as they progressed through the residency program. Senior faculty were also asked to comment on changes in overall resident performance and behavior from resident cohorts of previous years who did not participate in these new academic sessions. Three themes emerged:

1. Residents seemed better able to answer open-ended questions at the bedside and in the clinic.
2. Residents appeared more confident in interpreting clinical examination findings such as localizing lesions and distinguishing sometimes subtle normal from abnormal findings.
3. Senior faculty commented that most, but not all, residents participating in these new learning sessions appeared to perform better than their program-level counterparts from previous academic years, although these faculty indicated that other factors and experiences may have influenced resident performance.

## Discussion

### Planning Considerations

The effectiveness of efforts to integrate basic science content and faculty and into clinical education settings requires advanced planning. It is important that the specific needs and concerns of the clinical department be clearly defined and that the basic science department or departments providing educational activities have faculty that can make meaningful contributions. It is important that the basic science faculty members identified to participate in such collaborative effort can be freed from other academic activities in their home department without compromise to that department or to the individual.

For the efforts described here, the planning and approval processes involved the chairs of the neurology and anatomy departments. The contributions of the neurology department included specific guidance with regard to what was needed to address the concerns identified, chief of which was resident difficulty in describing the neuroanatomical underpinnings of the neurologic examination and the findings elicited. With this information clearly defined, specific content and teaching approaches were developed by the neurology program director and basic science faculty member and agreement was reached on related issues including how much time would be needed to address these topics. Importantly, scheduling and clinical coverage issues were addressed to permit resident attendance at the sessions without affecting their assigned clinical responsibilities. In this case, the sessions described were added to the weekly 3-h neurology department academic activities attended by all residents.

The anatomy department chair determined that appropriately trained faculty were available, that the project could be undertaken without negative effects on other teaching and scholarship responsibilities of the involved individual, and that tangible and meaningful benefits for the participating faculty could be realized. In addition, the neurology department chair suggested that the involved faculty member attend and participate in other resident learning activities including ward and grand rounds in an effort to better acquaint him with the specific needs of the residents. This opportunity was taken advantage of and was thought to be helpful in shaping specific content to be both included and excluded from the new sessions.

Payment or reimbursement for time and effort expended may also need to be considered. In some circumstances, where faculty salaries are secure, assignments of this type may be included in an annual assignment with no requirement to address salary issues. In some cases, the transfer of funds from one department to another may be possible. In situations in which this is not feasible, the department benefiting from the efforts may be able to provide funding for travel to professional meetings or offset cost related to the production of materials developed for other courses taught by the participating faculty. For the project described here, the instructor was not required to generate salary support from outside sources. However, for faculty without full faculty salary support, negotiations will be required to address this important factor. In addition, travel funds for the anatomy faculty to attend professional meetings were provided by the neurology department.

The instructional program described here included a decision to formally assess student progress annually and use this data for resident advising and for purposes related to program re-accreditation. The decision was made to administer this test at the beginning of each resident academic year to all residents and at completion of the final year of residency training. For first-year residents, test results provided a baseline from which to measure progress during the upcoming years. For residents further along in the program, examination data provided useful feedback for the resident and the program director. Thus, the time to develop, administer, grade, and review the examination results with the residents and program director was considered an important component of the project and was factored into an estimation of the time commitment required.

Cervantes et al. [8], Sakles et al. [9], and Wilkins et al. [10] have reported on successful efforts to incorporate basic science information and faculty into clerkship rotations. Students were found to benefit from the review and reinforcement of basic science content provided by the participating faculty. An additional benefit reported by these authors was increased basic science faculty awareness of the clinical learning environment and a greater appreciation of the role of the basic medical sciences in the clinical teaching setting. In some situations, as pointed out by Clay et al. [11], the nature of the participation of basic science faculty in clinical settings may need to be defined. Non-physician basic science faculty may be unfamiliar with issues related to patient confidentiality and may need guidance regarding their participation in patient-centered educational settings. Similar benefits for both faculty and learners could be envisioned by the participation of appropriate basic science faculty in other selected residency programs.

### Contributions to Program Development

Residency training is a period when newly graduated physicians further develop their knowledge, skills, and attitudes within their chosen specialty area. Progress includes not only further understanding of disease processes and their management, but also the basic science framework upon which diagnoses and treatment approaches are based. In neurology, a solid understanding of neuroanatomy in particular is central in localizing lesions disease processes, selecting and interpreting appropriate diagnostic tests, developing complete differential diagnoses, and establishing appropriate treatment plans. Effective collaborative relationships between neurology and basic science faculty with experience and expertise in the basic sciences critical to neurology can add substantively to program effectiveness and to the success of individual residents.

### Limitations

The educational program described here, intended to enhance the ability of neurology residents to succeed during training, was benefitted by the availability of an interested, experienced, and appropriately trained basic science faculty who could be made available to contribute to solving the concerns raised by the neurology department chair. An obvious limitation to the implementation of a similar program is the availability of such individuals at institutions where there may not be similarly trained basic science faculty. Equally important, the continuance of a such a program is dependent on the continued availability of participating faculty. Factors including faculty relocation to other schools and retirement represent sometimes unavoidable risks to the continuance of effective, interdisciplinary, collaborative efforts.

In addition, we acknowledge the difficulties associated with obtaining a reliable evaluative data regarding the success of our implemented learning activities. While the test scores obtained here may demonstrate trends in knowledge acquisition, we recognize that many learning opportunities exist in a residency program and that periodic written examination scores are not sufficient to demonstrate the effectiveness of one particular learning activity. Despite the challenges associated with evaluating the effectiveness of programs of this type, we strongly believe that efforts to measure effectiveness are necessary.

## Conclusion

Basic science faculty participation in the clinical teaching environment is one way in which program development and content integration can be achieved. We believe the program described here provided benefits to both departments, the participating faculty and residents involved. The approach described here is not limited to a particular basic science discipline or clinical specialty. The work described here was focused on an identified problem, helping residents with difficulties related to the understanding of the neuroanatomical underpinnings of the neurologic examination. However, opportunities for preclinical faculty involvement in clinical educational settings are not limited to this particular clinical task or this particular problem.

We note that significant planning is needed in order to develop and implement a program as extensive as the one described here. We also believe that efforts aimed at better integration among disciplines at both the preclinical and clinical levels are valuable and should be attempted wherever possible.
